# Daily activity in minimal footwear increases foot strength

**DOI:** 10.1038/s41598-021-98070-0

**Published:** 2021-09-20

**Authors:** Rory Curtis, Catherine Willems, Paolo Paoletti, Kristiaan D’Août

**Affiliations:** 1 Department of Musculoskeletal and Ageing Science, Faculty of Health and Life Sciences, University of Liverpool, Liverpool, UK; 2 Department of Design, KASK & Conservatorium, The School of Arts of HoGent and HoWest, Ghent, Belgium; 3 Department of Mechanical, Materials and Aerospace Engineering, School of Engineering, University of Liverpool, Liverpool, UK

**Keywords:** Musculoskeletal system, Biomechanics

## Abstract

The human foot is uniquely adapted to bipedal locomotion and has a deformable arch of variable stiffness. Intrinsic foot muscles regulate arch deformation, making them important for foot function. In this study we explore the hypothesis that normal daily activity in minimal footwear, which provides little or no support, increases foot muscle strength. Western adults wore minimal footwear for a six-month period (the “intervention” group). Foot strength, i.e., maximum isometric plantarflexion strength at the metatarsophalangeal joints, and foot biometrics were measured before and after the intervention. An additional group was investigated to add further insight on the long-term effects of footwear, consisting of Western adults with an average 2.5 years of experience in minimal footwear (the “experienced” group). This study shows that foot strength increases by, on average, 57.4% (p < 0.001) after six months of daily activity in minimal footwear. The experienced group had similar foot strength as the post intervention group, suggesting that six months of regular minimal footwear use is sufficient to gain full strength, which may aid healthy balance and gait.

## Introduction

The foot is usually the body’s only physical contact with the ground. Forces produced by the body are transmitted to the ground via the foot to generate forward propulsion in addition to supporting body weight^1^. The human foot has evolved a set of unique anatomical adaptations to support effective bipedal locomotion. Well-defined longitudinal arches had evolved by around 2 million years ago, found in early *Homo erectus*^2^. These adaptations help reduce midfoot motion, the latter shown as a pronounced “midtarsal break” in apes^3,4^ but present to some degree in humans^5^. The springy plantar aponeurosis present in modern day humans (*Homo sapiens*) reduces the cost of transport by cyclically storing and releasing energy during locomotion^6,7^. It is also considered a key component of the windlass mechanism^8^ which contributes to the foot’s ability to regulate stiffness^9,10^ but muscle action also contributes especially during push-off^11^. Humans also have considerable foot musculature which aids control the deformation of the foot’s arches^12,13^, helps stabilise the foot and improves balance during stance phase^14^.

Studies have shown that intrinsic foot muscles (such as the abductor hallucis, flexor digitorum brevis, and quadratus plantae^12,15^) actively influence longitudinal arch stiffness and elastic recoil^12,14–18^, in addition to passive contributions by the plantar aponeurosis. Therefore, strong intrinsic foot muscles may improve the longitudinal arch deforming mechanism, beneficial to an efficient gait and also (or even predominantly) to stiffen the distal foot joints during push-off^19^.

Increasing intrinsic foot muscle strength has been correlated positively with balance and stability, and may reduce fall risk in older people^20^. Conversely, weak feet have been shown to be a factor in fall risk^21^. Weak intrinsic foot muscles may also be associated with foot injury and deformities^22–26^ such as hallux valgus^27^, claw toe and hammer toe^28^. Given that strong intrinsic foot muscles improve stability and reduce foot deformities, stronger intrinsic foot muscles are desirable over weaker ones.

Foot muscle strengthening exercises are an effective way to strengthen the intrinsic muscles of the foot. Foot doming is an exercise that is commonly employed by clinicians to strengthen the foot, with much success^29^. Another potential method of foot strengthening might be the use of minimal footwear, defined as “Footwear providing minimal interference with the natural movement of the foot due to its high flexibility, low heel to toe drop, weight and stack height, and the absence of motion control and stability devices”^30^.

Studies have shown that foot strength can be increased by performing sports in flexible and minimal footwear^31–34^. However, this can also lead to injury if done excessively^35^. We hypothesize that low intensity activities of daily living (such as walking) in minimal footwear might increase foot strength too. Ridge et al.^36^ found that runners walking in minimal footwear for eight weeks increased their foot muscle strength. Holowka et al.^37^ found that the intrinsic foot muscles, abductor hallucis and abductor digiti minimi, were larger in a habitually minimally shod population than in a habitually conventionally western shod population.

In addition to strength and muscle size, minimal footwear may also influence foot morphology because they tend to have a wider toe box than conventional shoes. Habitually minimally shod participants have been found to have significantly higher longitudinal arches than conventionally western shod participants^38^. This is in agreement with a study by Hollander et al.^39^ who found significantly higher static arch heights in habitually barefoot children between the ages of six and 18 years when compared to their conventionally shod children. A study by D’Août et al.^40^ found no differences between static or dynamic longitudinal arch heights of habitually barefoot Indians, habitually shod Indians and conventionally western shod Europeans but, based on pressure recordings, the Indian groups had slightly more (albeit less variable) midfoot contact. In terms of morphology, most studies agree that habitually barefoot and/or minimally shod populations have wider feet^41–43^. This study will investigate minimal footwear influence on morphology as well as foot strength on habitually conventionally Western shod healthy adults.

This study has two central aims. The first is to quantify the influence of six months of regular minimal footwear use on foot strength and morphology, for adults that were previously habitually conventionally western shod. The second is to quantify the longer-term influence of regular minimal footwear use on foot strength and biometrics. We hypothesise the following:

1. Foot width increases in conventionally western shod populations after using minimal footwear for daily activity over a six-month period.
2. Foot strength increases in conventionally western shod populations after using minimal footwear for daily activity over a six-month period.
3. Foot strength is further increased in conventionally western shod populations if regular use of minimal footwear is longer than a six-month period.

## Methods

The present paper combines a prospective study and a cross-population study. The prospective study investigates the influence of six months of daily activity in minimal footwear on foot strength and biometrics, for adults that were habitually using conventional Western shoes prior to the study, and a control group. This group will be further referred to as the “intervention” study group. We further report on a group that has been studied cross-sectionally. This group consists of formerly conventionally shod western adults that have switched to minimal shoes for a longer period (2.5 ± 2.4 years). This group will be further referred to as the “experienced” study group.

### The “intervention” study

Habitually conventionally western shod participants transitioned from exclusively conventional footwear use to predominantly minimal footwear use for a six-month intervention period (n = 22, 13 male, 9 female, age 26.7 ± 6 years, BMI 24.4 ± 2.7). We also measured a control group consisting of similar participants, but which continued to wear their own conventional footwear throughout the six-month period (n = 24, 14 male, 10 female, age 28.4 ± 7.4 years, BMI 22.8 ± 3.1). The number of participants was limited by the ability to recruit, retain, and measure the participants in this relatively demanding longitudinal study. All participants had morphology, basic biometrics and foot strength measured before and after the 6-month period. Intervention and control sub-groups’ biometrics and foot strength matched well before the study started (Table 1).

**Table 1:** Biometrics and activity patterns of the intervention group pre and post intervention period, split into control and intervention sub-groups. “Reported weekly activity” and “Weekly reported footwear use” range over both pre and post intervention columns as these characteristics were taken during the six-month intervention period.

Biometric or activity	Control (n = 24)		Intervention (n = 22)	
	Pre	Post	Pre	Post
Age (yrs)	28.4 ± 7.5	28.9 ± 7.5	26.7 ± 6.2	27.3 ± 6.2
Mass (kg)	67.7 ± 11.9	67.6 ± 11.5	73.2 ± 12.8	73.1 ± 11.8
Height (cm)	172.2 ± 6.3	172.9 ± 5.5	172.7 ± 8.3	173.8 ± 8.3
BMI	22.7 ± 3.1	22.5 ± 2.8	24.4 ± 2.8	24.1 ± 2.7
Leg length (mm)	912 ± 41	906 ± 34	904 ± 48	900 ± 53
Foot length (mm)	252 ± 13	251 ± 17	252 ± 17	251 ± 17
Foot width (mm)	95.6 ± 5.3	94.8 ± 4.8	99.6 ± 8	99.3 ± 8
Toe length (mm)	68 ± 5.6	68 ± 3.9	69 ± 9.7	68 ± 5.2
Navicular height (mm)	48 ± 7.4	48 ± 6.7	49 ± 7.3	46 ± 5.1
Reported weekly activity (hrs)	31.3 ± 20.8	25 ± 25.1		
Weekly reported footwear use (hrs)	49.2 ± 17.3	52.7 ± 17.3		

**Figure 1:**
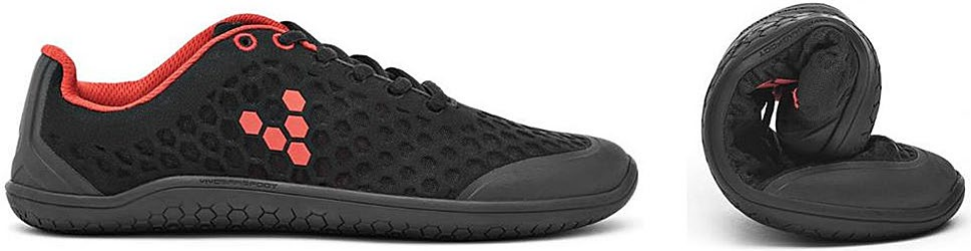
Lateral views of the minimal footwear used in the intervention study (Vivobarefoot Stealth II). Image: Vivobarefoot (with permission).

The intervention (i.e., non-control) sub-group were given minimal footwear (Vivobarefoot Stealth II; Fig. 1) to wear for the intervention period. They were required to wear this footwear for a minimum of 70% of the time they were shod, and at least six days per week. Control participants followed the same constraints for their most frequently worn conventional footwear. In addition to this, intervention participants were informed of the possible risks of running in minimal footwear and were instructed not to use the shoes for such activities.

Participants were only recruited if they met the following inclusion criteria: free from lower limb pathologies for a minimum of six months prior to the start of the study, aged between 18–55 years, a BMI between 18.5–30, and had never worn minimal footwear before.

All participants filled out a weekly activity log throughout the intervention period to monitor activity, footwear wearing patterns and compliance. This was also used as the platform to communicate any discomfort. Participants finished the study within the six-month intervention period ± one week, except for one participant who finished twelve days after the intended end date. Five participants dropped out due to injury (unrelated to the study) or failure to keep up with the study requirements. These participants are not reported within this study and are not included in the numbers mentioned above.

All data for the intervention study was collected in the gait laboratory at the University of Liverpool under ethics approval granted by the University of Liverpool Health and Life Sciences Research Ethics Committee (Human participants, tissues and databases), reference number 1911. All methods were performed in accordance with the relevant guidelines and regulations.

At the start of the study, participants came into the laboratory and filled out the informed consent form and an activity, health, and footwear habits questionnaire. They then changed into non-restrictive clothing and had biometrics recorded. Body mass and height were measured with a Seca 360 clinical-grade scale (e = 0.05 kg). Foot length was measured using a flat metal ruler under the participants’ feet. The participants then stood up straight with the feet apart at shoulder width, and foot length was measured as the distance between the most posterior point of the heel to the most distal point of the most distal toe (either the hallux or the 2nd toe). Foot width was measured from the medial side of the 1st metatarsal to lateral side of the 5th metatarsal heads using digital callipers (e = 0.1 mm). Toe length was measured as the length of the hallux, from the centre of the first metatarso-phalangeal joint to the most distal part of the hallux. Navicular height (a measure for static arch height) was measured from the palpated centre of the navicular tuberosity to the ground using a tape measure, while weight bearing. Leg length was measured from the palpated centre of the greater trochanter of the femur to the ground using a tape measure. These biometrics were measured before and after the intervention period (Tables 1 and 2). Relative arch height was calculated as the navicular height divided by truncated foot length (i.e., foot length minus hallux length) as a proxy for medial longitudinal arch length.

**Table 2:** Biometric and statistical comparisons between the study groups.

Biometrics	Intervention (n = 46)	Experienced (n = 20)	P
Age (years)	27.6 ± 6.9	31.05 ± 7.1	0.20
Mass (kg)	70.3 ± 12.5	68.6 ± 9.4	0.83
Height (cm)	172.4 ± 7.3	173.5 ± 9.8	0.87
BMI	23.5 ± 3	22.8 ± 2.9	0.69
Leg Length (mm)	908 ± 44	933 ± 52	0.12
Foot Length (mm)	252 ± 15	255 ± 16	0.42
Foot Width (mm)	97 ± 6.9	98 ± 7.8	0.76
Toe Length (mm)	69 ± 8	70 ± 7	0.71
Navicular Height (mm)	48 ± 7	53 ± 7	**0.03**

Significance level is set at P < 0.05.

Prior to their arrival at the laboratory, participants were instructed to bring the footwear they most regularly wore for the initial Gait Lab study. The brand, name and shoe size of each participant’s footwear was recorded. The regular footwear was then weighed using OHaus Scout weighing scales (e = 0.1 g). Shoe length was the linear distance from the back of the footwear’s heel to its most distal tip. Shoe width was the linear distance from each end of the widest point of the footwear sole (using digital callipers). Sole thickness was the thickness from the central part of the heel section to the base of the sole by using outside callipers. Stack height was calculated as in Eq. (1).

1 $$stack\;height = sole\;thickness - \left( {toe\;box\;thickness - upper\;thickness} \right)$$

Toe box thickness was the thickness of the sole of the central toe box area as well as the upper thickness above it when the upper material was manually pushed down. Upper thickness was measured as the upper material thickness directly above the centre of the toe box area using callipers. The right shoe of each participant’s regular footwear was then placed in a specialised jig fitted to a Lloyd LRX worm drive material property tester. Tests were performed to quantify the footwear’s bending stiffness by measuring the force required to bend the footwear to 25 degrees about the MPJ region, with an applied moment arm of 5 cm, and its sole stiffness. The spatial and mechanical properties of the minimal footwear were also measured following the same procedure. A men’s 41 EU Vivobarefoot Stealth II shoe was used to take the footwear properties from, corresponding to the average foot length of the participants of 252 mm. The minimal footwear’s spatial properties were measured once and mechanical properties were measured five times, and the average was taken. The footwear properties are shown in Table 4.

**Figure 2:**
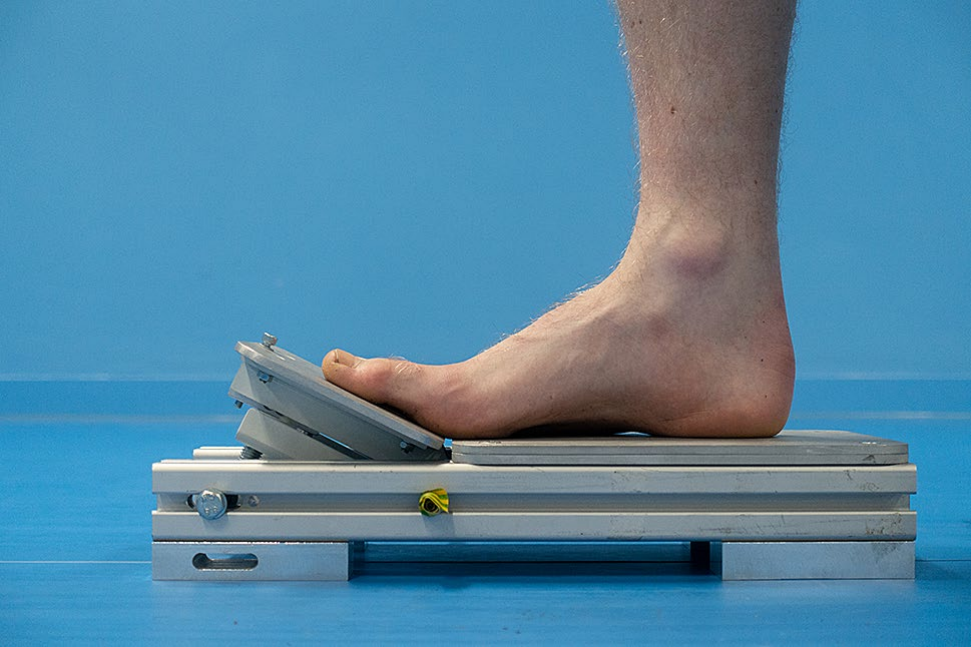
Image showing foot position on the dynamometer.

Finally, participants had foot strength tested. This was done using a modified version of a technique employed and validated by Goldmann et al.^32,44,45^. This method quantifies foot strength as the maximum isometric plantar flexion strength of the toes about the metatarso-phalangeal joint. For the purposes of this study this measure of foot strength will be referred to as Toe Flexion Strength (TFS). In order to measure TFS, a custom dynamometer was built which recorded the moment (Nm) generated by TFS at a sample frequency of 4.9 Hz and accuracy of 0.1 Nm. During this study the load plate of the device was angled to 25°. This value was chosen as Goldmann et al.^32^ found this angle to be successful in showing changes in TFS before and after athletic exercising in minimal footwear. Participants were instructed to sit on an adjustable chair with the back straight and flush with the back rest. Their right bare foot was then placed onto the dynamometer, taking special care to correctly position the metatarsophalangeal joint at the device’s plate division so that the hallux and lesser toes rested on the angled load plate (Fig. 2). The participant’s position was adjusted until the investigator was satisfied that the participant’s knee and ankle angle were both at 90° upon visual inspection. The participant was instructed to push as hard as they could with their toes onto the load plate while making sure to keep their heel on the base plate. They were instructed to keep their back straight, taking care not to lean back into the back rest of the seat. Participants were allowed practice attempts as required to reliably repeat the task and reach a plateau in the output. Participants were given a minimum of one minute of rest after the practice trails before going into the test. Participants completed five trails, each lasting 10 s with one minute of rest between trials.

### The “experienced” study

Experienced Western minimally shod walkers, but who had transitioned from a conventionally shod background were recruited if they have been using minimal footwear as their most frequently worn footwear for at least six months prior to starting the study. All these participants were recruited from the UK (n = 20, 10 female, 10 male, age 31.1 ± 6.7 years, BMI 22.8 ± 2.7). Subject numbers were limited by our ability to recruit and measure them outside of the laboratory, using a similar methodology to that of the intervention study. All data for the “experienced” study was collected under ethics approval granted by the University of Liverpool Health and Life Sciences Research Ethics Committee (Human participants, tissues, and databases), reference number 1911. All methods were performed in accordance with the relevant guidelines and regulations. Participants had their biometrics (Table 1), footwear properties (Table 4) and TFS recorded using the same methods employed in the “intervention” study. The material properties of the shoes were not recorded, but all shoes were the same brand, but of a variety of slightly different models as the minimal shoes used in the intervention study. Participants filled out the questionnaire and indicated how long they had been regularly wearing minimal footwear for.

### Analysis and statistics

The dynamometer data was recorded as text files and imported into Matlab2017a (MathWorks, Natick, MA, USA). All data were then smoothed with a low-pass 2nd order Butterworth filter set at 1 Hz to remove transient spikes that were not representative of the force plateau reached. The maximum moment was taken from each trial and the average maximum moment was calculated for each participant.

For the intervention study, change in TFS was calculated, per subject, as the percentage change in TFS post intervention compared to the pre-intervention baseline, as in Eq. (2):

2 $$Change\;in\;TFS = \frac{{TFS_{post} - TFS_{pre} }}{{TFS_{pre} }} \times 100$$

For the biometrics data, the normally distributed variables were analysed using Repeated Measures two-way ANOVA (factor group: control or intervention and factor time: pre or post) with subject as a random factor for the Intervention study. The biometric variables that were not normally distributed (Shapiro–Wilk test and visual inspection of histograms) in the Intervention study were Age (not tested as the subjects aged 6 months during the intervention, by definition) and Reported Weekly Activity. Reported weekly Activity was tested using a Mann–Whitney U test. In the Experienced group, Activity, Age, Foot length and Height were not normally distributed and tested with a Mann–Whitney U test for the unpaired data. For the other comparisons, two-tailed unpaired t tests were used. Separate comparisons were run between the pre and post conditions in the Intervention study, and between the subjects of the Intervention study and those of the Experienced study. The biometrics tests were run in R 4.0.2.

Footwear measurements were normally distributed and compared between groups with unpaired two-tailed t-tests (for two-group comparisons) or one-way ANOVAs and Tukey–Kramer post-hoc tests (for three-group comparisons) in MatLab 2017a.

TFS was not normally distributed and Mann–Whitney U tests were used for comparisons between the change in foot strength between control and intervention groups. For comparisons between the Intervention pre, Intervention post, and Experienced subjects, TFS was normalised to body mass. TFS per unit mass for each population had a non-normal distribution and was analysed using a Kruskal–Wallis test for the overall effect and Mann–Whitney U tests with Bonferroni corrections for the pairwise comparisons in SPSS 25.0.

## Results

### Biometrics

Biometrics for the intervention study participants (control and intervention sub-groups) are shown in Table 1. Repeated measured two-way ANOVA showed no interaction effect between group (control or intervention) and time (pre or post) for any of the variables. The only comparisons that reached significance were foot width between groups (approx. 4 mm; p = 0.03) and height between pre and post measurements (approx. 1 cm; p = 0.001).

Biometric data and comparisons between both studies are shown in Table 2. T tests and Mann–Whitney U tests revealed no significant differences between the populations except for navicular height, which was greater, by 5 mm, in the Experienced population.

Relative arch height was not significantly different between control and intervention groups, nor between pre and post intervention conditions in each group in the Intervention study (control-pre: 0.259 ± 0.038; control-post: 0.26 ± 0.037; intervention-pre: 0.269 ± 0.044; intervention-post: 0.253 ± 0.029). In the Experienced study, relative arch height was significantly (p = 0.035) higher (0.287 ± 0.04) than in all groups from the Intervention study.

### Footwear properties

Table 3 shows footwear spatial and material properties of the minimal footwear given to the intervention sub-group, the conventional footwear worn by the control sub-group of the intervention study, and the footwear worn by the experienced participants on the day of testing. Table 4 shows the comparisons between these types of footwear. One-way ANOVA showed significant differences. The intervention group footwear and the experienced group footwear were very similar, with upper thickness being the only significant difference. The conventional western footwear brought in by the control participants in the intervention study was highly variable and was significantly different from the intervention footwear in all attributes tested, except for shoe length. As expected, minimal shoes were lighter and wider than conventional shoes, with thinner, less compliant soles with a smaller bending stiffness.

**Table 3:** Spatial and material properties of the footwear used in the intervention study (INT), the ‘conventional’ footwear (CON) worn by the intervention study participants before they took part in the study, and the minimal footwear worn by the experienced participants on the day of testing (EXP).

Footwear properties	INT (n = 5)	CON (n = 46)	EXP (n = 20)
Sole thickness (mm)	5	32.6 ± 44.7	7.9 ± 4.4
Upper thickness (mm)	0.5	3 ± 1.6	1.5 ± 1.2
Sole offset (mm)	0	12.2 ± 8.5	0.2 ± 4.6
Shoe length (mm)	284	285 ± 20	275 ± 24
Shoe width (mm)	106.7	101.5 ± 6.7	104.4 ± 9.4
Shoe mass (g)	202	350 ± 105	199 ± 38
Bending force (N)	5.48 ± 0.16	13.25 ± 6.17	N/A
Sole compliance (mm/N)	0.022 ± 0.003	0.079 ± 0.031	N/A

**Table 4:** Spatial and material properties: test results of the statistical comparison between the footwear used in the intervention study (INT), the “conventional” footwear worn by all intervention study participants but before they took part in the study (CON) and the footwear worn by the experienced participants on the day of testing (EXP).

Biometrics	INT versus CON	INT versus EXP	CON versus EXP
**p-values for footwear properties comparisons between the three groups**			
Sole thickness	** < 0.001**	0.48	** < 0.001**
Upper thickness	** < 0.001**	0.03	** < 0.001**
Sole offset	** < 0.001**	0.1	** < 0.001**
Shoe length	0.97	0.27	0.11
Shoe width	**0.013**	0.51	0.25
Shoe weight	** < 0.001**	0.99	** < 0.001**
Bending force	** < 0.001**	–	–
Sole hardness	** < 0.001**	–	–

Significance level is set at P < 0.05.

### Participant history

Information gathered from the questionnaire is summarised in Table 5. Weekly activity and footwear age for the “intervention” and “experienced” groups were not significantly different before the start of the study. Weekly use of the group’s respective footwear was significantly higher in the experienced group than the intervention group, before the start of the study.

**Table 5:** Participant and footwear history comparisons between the total intervention study group, but before the study, and the experienced group. p-values of < 0.01 or < 0.001 are represented by ’**’, and ‘***’ respectively. Regular footwear is the actual footwear the subjects wore most regularly when they started the study. Footwear type is the type design of the footwear (e.g., trainers, dress shoes, etc.).

Footwear use and activity	Intervention group (n = 46)	Experienced group (n = 20)
General Activity per week (hours)	28.3 ± 22.9	38.7 ± 33.1
Regular footwear age (years)	1.1 ± 0.8	1.5 ± 1.3
Time spent in regular footwear type (years)	8.8 ± 6.3***	2.5 ± 2.4***
Weekly use of regular footwear (hours)	50 ± 16.8**	70.2 ± 25.2**

### Toe flexion strength

**Figure 3:**
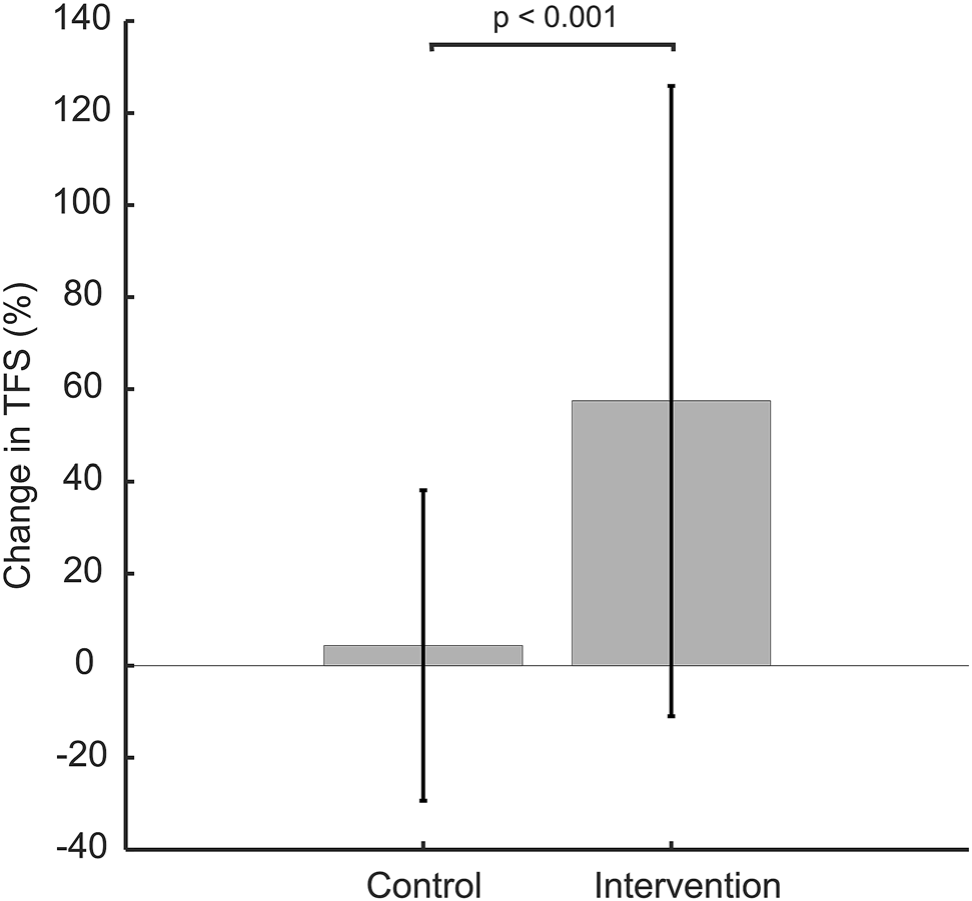
Percent change in “Toe flexion strength” (TFS, Eq. 2) at the end of the intervention study compared to the start.

The change in TFS in the intervention study is shown in Fig. 3, for both the intervention and control sub-groups. There is no significant change in TFS for the control group but there is a significant change in TFS for the intervention group (57.4 ± 68.4%, p = 0.000). The effect size of the change in TFS of the intervention group was calculated as Cohen’s “d” value of 0.84, representing a large effect.

**Figure 4:**
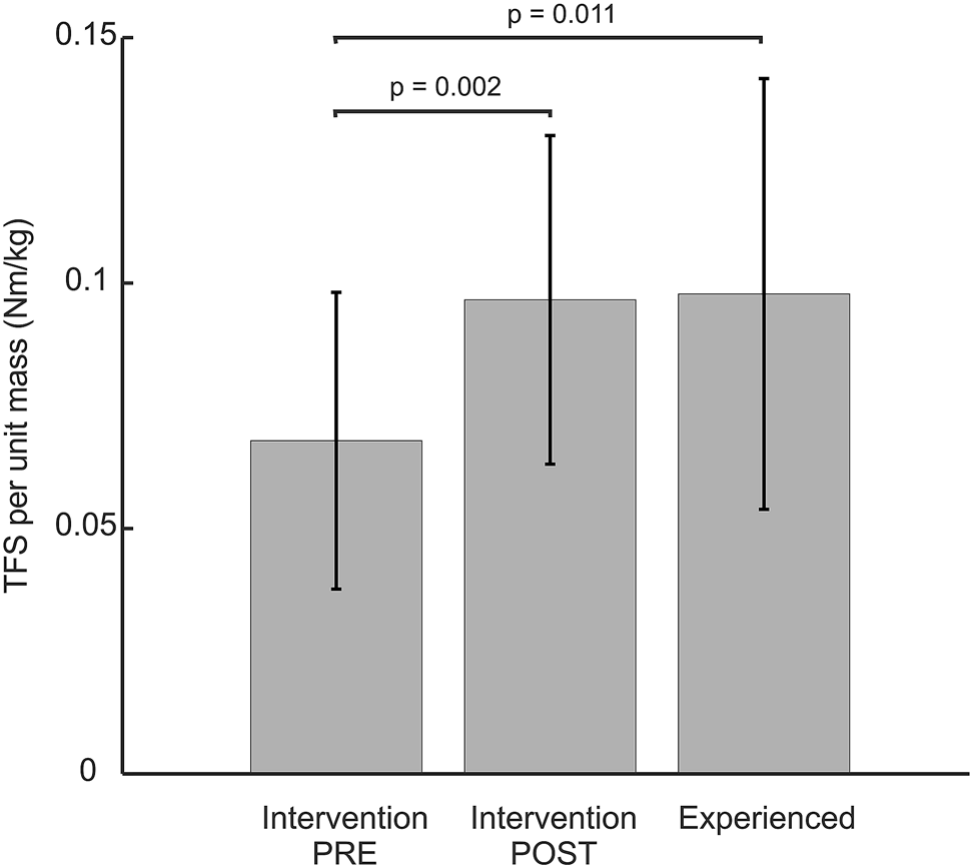
Toe flexion strength normalized to body mass and then averaged for the study groups. Left to right: intervention group at the start of the study (“pre”), intervention group at the end of the study (“post”), experienced study group.

For the cross-population comparison, TFS was normalised to body mass and is shown in Fig. 4. The three groups differ significantly (p = 0.005); more specifically after the 6-month period in the intervention study, the intervention sub-group obtained a significantly (p = 0.002) increased TFS per unit mass. The experienced group also had a significantly greater TFS per unit mass (p = 0.011) compared to the pre-intervention baseline values of the intervention group.

## Discussion

Our first hypothesis was that the foot would increase in width after switching to minimal shoes for six months. As can be seen from Table 1, we are rejecting this hypothesis. The foot width of the experienced group was also not significantly different than that of the other Western participants. The feet of the Control group were approximately 4 mm wider than those of the Intervention group, but both groups did not change during the study. It is important to mention that we measured width only at the level of the metatarsal heads (ball of the foot) and it is possible that other areas of the foot have changed. The lack of change of ball width may be due to foot plasticity of adults being much lower than that of children^46^. This could explain why habitually barefoot and or minimally shod populations, where people have not worn conventional Western shoes from childhood, have wider feet than Western shod populations^41–43^. Even though ball width did not increase as a result of six months (intervention group) or more (experienced group) of regular minimal footwear use, we hypothesise, based on anecdotal evidence, that toe splay might increase, and we suggest that future studies investigate this.

The intervention group’s absolute and relative navicular height, as measures for longitudinal arch height were similar pre and post intervention period. However, the experienced group’s navicular height was significantly higher that of the intervention group prior to the 6-month intervention. This suggests that regular minimal footwear use for periods of time greater than six months will increase static longitudinal arch height. The current literature on longitudinal arch height is conflicting. Some studies found habitually barefoot and/or indigenously minimally shod participants had greater static longitudinal arch height than habitually conventionally western shod subjects^38,46^, whereas D’Août et al.^40^ found no differences in longitudinal arch height. However, they noted that the variation in longitudinal arch heights was less variable in the habitually barefoot group and much less variable than in the conventionally shod Western group. This suggests that the latter group is more prone to extreme foot morphologies and conditions like pes planus (flat foot). It will be interesting to investigate in the future if the observed arch height increases after wearing minimal shoes is due to a uniform increase in all subjects, or due to an increase specifically in the lowest-arched individuals. Arch height is a crude measure for arch stiffness, and future research should assess how this changes as a result of minimal footwear.

The key finding of this study is that wearing minimal shoes for six months, even for non-intensive daily activities, increases toe flexion strength by 57.4% in a general population (Fig. 3). This is in alignment with the research by Ridge et al.^36^, who studied experienced runners and observed an 41% increase after eight weeks of walking in minimal footwear. We used an extended period of six months, and also compared the results from this intervention study with those of long-time minimal footwear users. As regular use of minimal footwear was the only intervention introduced in our study, and the control group showed no changes, it must be concluded that daily activity in minimal footwear increases foot strength for healthy adults, confirming our second hypothesis. Mechanistically, this is probably due to the lower bending stiffness of minimal shoes compared to conventional shoes, which are typically harder to flex about the MPJ, and may also be part explained by the absence of a structural toe spring which has been suggested to weaken the foot^47^. This stiffness contributes to the resistive force required for the foot to be a stiff lever upon push-off, thereby reducing the demand on the foot muscles during gait. Over time this will ultimately lead to a loss of mass in the foot muscles, much like the muscle atrophy experienced by muscles immobilised by body casts^48^, but to a lesser extent.

It should be noted that the TFS we measured is most likely a combination of both intrinsic and extrinsic foot muscles, but both share several functions^29^. We have not used MRI or ultrasound imaging to quantify the size of individual muscles^36,37^. Figure 4 shows that previously Western adults with at least six months experience in minimal footwear have greater TFS per unit mass than their peers with no minimal footwear experience. In addition to this, TFS per unit mass of the former individuals with just six months experience of regular minimally shod walking is very similar to those with longer (in this study, on average 2.5 years) of minimally shod walking experience. This rejects our third hypothesis that foot strength would further increase after 6 months. Thus, the comparison of our Intervention study and our Experienced study suggests that six months of using minimal footwear on a regular basis may be a sufficient time to rehabilitate the foot muscles, but eight weeks would be too short as it yields a ~ 40% increase^36^.

Obtaining a stronger foot might have several benefits. It could be important as a preparation for individuals wishing to run in minimal footwear. Several previous studies have shown that minimally shod running can increase the risk of injury^35,49^. All of these studies started with participants with no or very little experience with minimal footwear. Injury free minimally shod running may be possible once sufficient foot strength is reached. However, it should be noted that muscular foot strength is only one aspect of foot function. The bones of the foot require sufficient time to strengthen as well. Increased mechanical loading on the bone promotes bone growth^50^. Therefore, regular walking in minimal footwear may be more beneficial than just foot strengthening exercises on its own as and it may strengthen the foot bones as well as the muscles. We suggest that one must walk before they can run when it comes to minimally shod locomotion.

Important health benefits can potentially be gained by increasing foot strength, which is likely to reduce the chance of developing foot deformities associated with weak intrinsic foot muscles such as hallux valgus^27^, claw toe and hammer toe^28^. Additionally, intrinsic foot muscle strength is positively correlated to stability^51^. It has also been shown that increasing intrinsic foot muscle strength positively influences balance and stability, and reduces fall risk in older people^20^. This is of particular importance as nearly one third of older people experience at least one fall a year^52^ impacting on their quality of life.

To conclude, toe flexion strength increases by nearly 60% after using minimal shoes for daily activities for six months. Achieving greater foot strength can have multiple benefits, including athletic performance, foot health, and an improvement in gait and risk of falls.
